# Ocular pulse amplitude (OPA) in canine *ADAMTS10*-open-angle glaucoma (*ADAMTS10*-OAG)

**DOI:** 10.3389/fbioe.2023.1242166

**Published:** 2023-12-07

**Authors:** Vanessa A. Raphtis, Dhruv Sharma, Sichao Wang, Jae Y. Kim, Amanda L. Jacobson, Christine D. Harman, András M. Komáromy

**Affiliations:** ^1^ Department of Small Animal Clinical Sciences, College of Veterinary Medicine, Michigan State University, East Lansing, MI, United States; ^2^ Center for Statistical Training and Consulting, Michigan State University, East Lansing, MI, United States

**Keywords:** *ADAMTS10*, dog, glaucoma, intraocular pressure (IOP), ocular pulse amplitude (OPA), ocular rigidity

## Abstract

**Introduction:** The role of ocular rigidity and biomechanics remains incompletely understood in glaucoma, including assessing an individual’s sensitivity to intraocular pressure (IOP). In this regard, the clinical assessment of ocular biomechanics represents an important need. The purpose of this study was to determine a possible relationship between the G661R missense mutation in the *ADAMTS10* gene and the ocular pulse amplitude (OPA), the difference between diastolic and systolic intraocular pressure (IOP), in a well-established canine model of open-angle glaucoma (OAG).

**Methods:** Animals studied included 39 *ADAMTS10*-mutant dogs with different stages of OAG and 14 unaffected control male and female dogs between 6 months and 12 years (median: 3.2 years). Dogs were sedated intravenously with butorphanol tartrate and midazolam HCl, and their IOPs were measured with the Icare^®^ Tonovet rebound tonometer. The Reichert Model 30™ Pneumotonometer was used to measure OPA. Central corneal thickness (CCT) was measured via Accutome^®^ PachPen, and A-scan biometry was assessed with DGH Technology Scanmate. All outcome measures of left and right eyes were averaged for each dog. Data analysis was conducted with ANOVA, ANCOVA, and regression models.

**Results:** *ADAMTS10*-OAG-affected dogs displayed a greater IOP of 23.0 ± 7.0 mmHg (mean ± SD) compared to 15.3 ± 3.6 mmHg in normal dogs (*p* < 0.0001). Mutant dogs had a significantly lower OPA of 4.1 ± 2.0 mmHg compared to 6.5 ± 2.8 mmHg of normal dogs (*p* < 0.01). There was no significant age effect, but OPA was correlated with IOP in *ADAMTS10*-mutant dogs.

**Conclusion:** The lower OPA in *ADAMTS10*-mutant dogs corresponds to the previously documented weaker and biochemically distinct posterior sclera, but a direct relationship remains to be confirmed. The OPA may be a valuable clinical tool to assess ocular stiffness and an individual’s susceptibility to IOP elevation.

## Introduction

Glaucoma is a common, incurable neurodegenerative disease and the leading cause of irreversible blindness worldwide, affecting over 70 million people (Quigley and Broman, 2006). Open-angle glaucoma (OAG) and angle-closure glaucoma are the two primary disease types, depending on the particular abnormalities of the aqueous humor outflow pathways within the iridocorneal angle that increase intraocular pressure (IOP) (Weinreb et al., 2014; Jonas et al., 2017). The pathogenic mechanisms of glaucomatous optic neuropathy leading to retinal ganglion cell (RGC) death and vision loss remain only partly understood. The three primary risk factors are advancing age, genetics, and IOP-related biomechanical stress on the optic nerve head (ONH) (Weinreb et al., 2016; Jonas et al., 2017). Current glaucoma treatment is limited to lowering IOP by medical and surgical means (Weinreb et al., 2014; Jonas et al., 2017).

Even though the prevalence of glaucoma increases with IOP elevation, glaucomatous ONH damage can develop at any IOP level based on significant interindividual variabilities in susceptibility (Sommer et al., 1991; Weinreb et al., 2016). At diagnosis, approximately half of primary OAG (POAG) patients have normal IOP between 10–20 mmHg (Collaborative Normal-Tension Glaucoma Study Group, 1998a; Koz et al., 2009; Weinreb et al., 2016). Nevertheless, IOP-related biomechanical stress is important in these normal tension glaucoma (NTG) patients because a 30% IOP reduction significantly slowed disease progression (Collaborative Normal-Tension Glaucoma Study Group, 1998b). In contrast, individuals with ocular hypertension (OHT) can tolerate increased IOP >21 mmHg without ONH damage (Weinreb et al., 2016). Susceptibility to IOP may be based on connective tissue properties of the fibrous tunic of the globe consisting of cornea, sclera, and lamina cribrosa of the ONH (Bellezza et al., 2000; Burgoyne et al., 2005; Downs et al., 2009; Burgoyne, 2011; Park and Komaromy, 2021).

The biomechanical properties of the eye have been studied in great detail in the laboratory setting using human cadaver eyes and animal models (Coudrillier et al., 2016; Hopkins et al., 2020; Park and Komaromy, 2021; Safa et al., 2022). Clinical measurements of connective tissue properties in human glaucoma eyes are limited to the cornea and include central corneal thickness (CTT) and corneal hysteresis by use of Ocular Response Analyzer^®^ (ORA) (Gordon et al., 2002; Medeiros et al., 2013; Ayyalasomayajula et al., 2016; Weinreb et al., 2016). These measurements exclude the posterior pole of the globe, including the peripapillary sclera (**PPS**) and the lamina cribrosa, whose biomechanical factors likely impact the ONH’s IOP susceptibility (Downs et al., 2009; Burgoyne, 2011; Safa et al., 2022). There is a critical need for additional clinical tools to measure ocular biomechanics that are not limited to the cornea to evaluate susceptibility to IOP in patients.

In this study, we tested the hypothesis that the measurement of ocular pulse amplitude (OPA) may allow the comparison of biomechanical properties of the entire fibrous tunic *in vivo*. The OPA is defined as the IOP difference between systole and diastole of the pulsatile choroidal blood flow, and appears to be correlated with ocular rigidity based on observations in glaucoma patients (Tonjum, 1972; Dastiridou et al., 2009). We took advantage of a well-defined, clinically relevant canine glaucoma model, the OAG-affected Beagle, with a G661R missense mutation in the *ADAMTS10* gene (Kuchtey et al., 2011). Differences in IOP susceptibilities are well recognized by veterinary ophthalmologists when comparing glaucoma-affected dog breeds (Park and Komaromy, 2021). For example, Beagles with *ADAMTS10*-OAG tolerate elevated IOP much better, with slower progression of vision loss, than other glaucoma-affected breeds with comparable IOPs (Kuchtey et al., 2011; Park and Komaromy, 2021). Compared to normal dogs, the posterior scleral of *ADAMTS10*-mutant Beagles is biochemically distinct and weaker with a lower normalized ocular rigidity (Palko et al., 2013; Boote et al., 2016; Palko et al., 2016). This study shows a significantly smaller OPA in *ADAMTS10*-mutants whose sclera is softer than the normal, supporting the potential clinical value of OPA as a measure of glaucoma susceptibility.

## Materials and methods

### Animals

**FIGURE 1:**
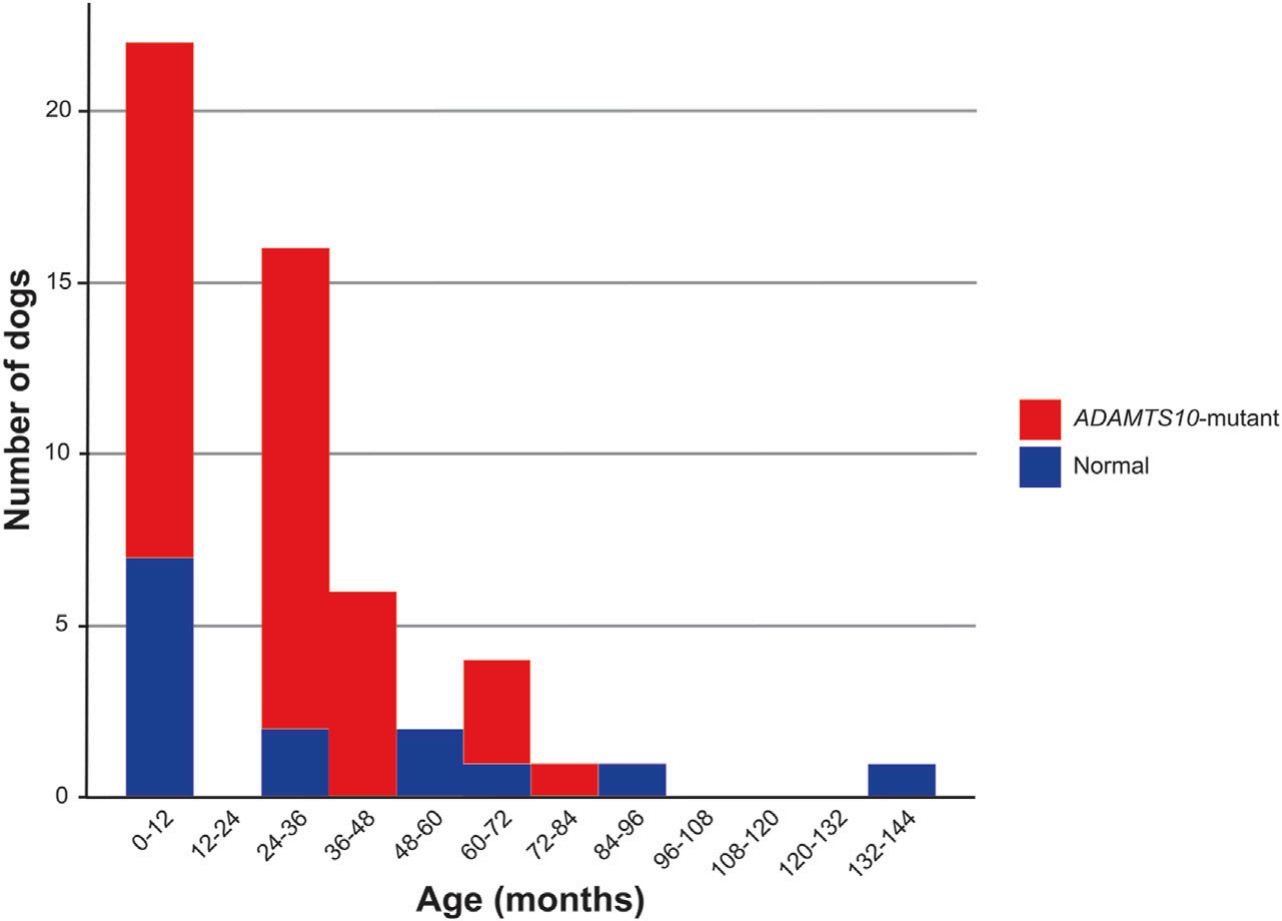
Age distribution. This histogram compares the age distribution for normal and *ADAMTS10*-mutant dogs enrolled in the study.

A total of 101 eyes of 53 purpose-bred dogs (both eyes of 48 dogs and one eye of 5 dogs each), 39 at different stages of *ADAMTS10*-OAG, and 14 normal, glaucoma-unaffected dogs were used. Twenty-six males and 27 females were included between the ages of 6 months and 12 years (median: 3.2 years) (Figure 1). In addition, a separate independent experiment was performed to determine the repeatability of OPA measurements in dogs: A total of 34 eyes of 18 purpose-bred dogs (both eyes of 16 dogs and one eye of 2 dogs each), 14 at different stages of *ADAMTS10*-OAG, and 4 normal, glaucoma-unaffected dogs were used. Ten males and 8 females were included between the ages of 1–8 years (median: 2.8 years).

Genotypes were confirmed based on the *ADAMTS10* gene sequence: glaucomatous dogs were homozygous for the G661R missense mutation, which is responsible for OAG in Beagles, while the normal dogs were either carriers of the mutation or homozygous for the wildtype allele (Kuchtey et al., 2011). Glaucomatous dogs that received pressure-lowering medication were excluded from this study. The dogs were group-housed in the same environment at the Michigan State University College of Veterinary Medicine with a 12 h/12 h light/dark cycle and fed the same diet.

### Study design

Dogs were sedated intravenously with a combination of 0.3 mg/kg butorphanol tartrate (Bayer Healthcare LLC, Animal Health Division, Shawnee Mission, KS, United States) and 0.3 mg/kg midazolam HCl (Avet Pharmaceuticals Inc., East Brunswick, NJ, United States). Dogs were placed in a lateral recumbent position for blood pressure measurements. Systolic, diastolic, and mean arterial blood pressures were measured with Mindray Passport^®^ 12 Patient Monitor and blood pressure cuff (Mindray, Shenzhen Mindray Bio-Medical Electronics Co., Ltd., Nanshan, Shenzhen, Guangdong Province, China) placed over the left or right dorsal pedal artery. Intraocular pressure was measured manually via iCare TonoVet^®^ rebound tonometer (Icare Finland Oy, Vantaa, Finland) while dogs were in a seated position. Mean perfusion pressure (PP) was calculated as the difference between mean arterial blood pressure and IOP, and vascular pulse amplitude (VPA) as the difference between systolic and diastolic blood pressure.

**FIGURE 2:**
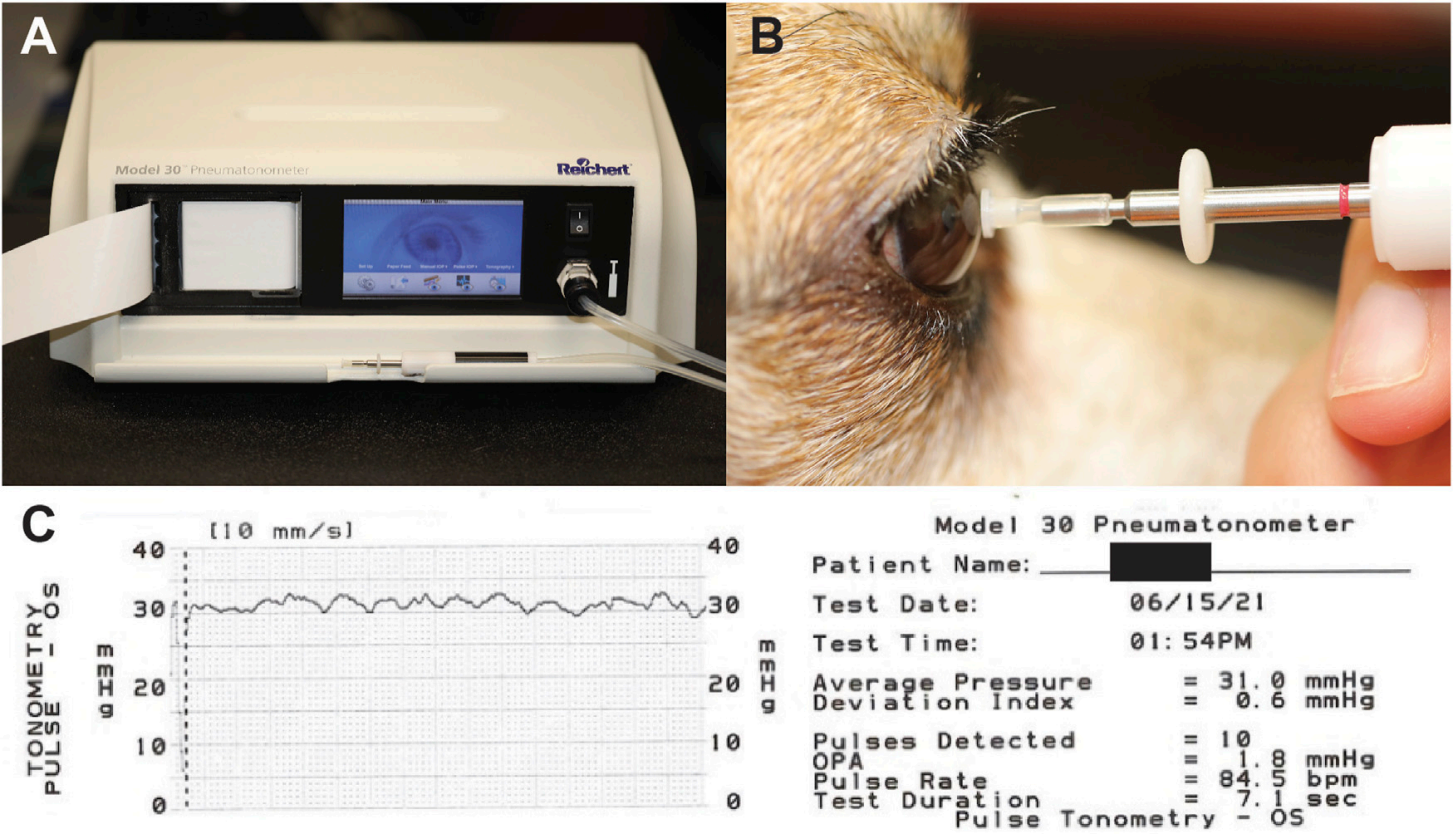
Equipment and setup for OPA measurements. **(A)** The Reichert Model 30™ Pneumotonometer. **(B)** Placement of pneumotonometer probe on central corneal. **(C)** Chart produced by Reichert Model 30™ Pneumotonometer documenting measurements recorded.

The left or right eye was randomly selected for every dog to begin measurements for OPA. Dogs were placed in sternal position for pneumotonometer measurements. Ocular surface anesthesia was achieved by administering one drop of proparacaine HCl 0.5% ophthalmic solution (Akorn, Inc., Lake Forest, IL, United States). The Reichert Model 30™ Pneumotonometer (Reichert, Inc., Depew, NY, United States) was used to measure OPA as well as provide additional measurements of IOP, a deviation index, and pulsation rate (Figure 2A). Measurements were recorded by placing the pneumotonometer probe tip on the central cornea following manufacturer guidelines (Figure 2B). The probe tip contains a small (5-mm diameter) fenestrated membrane that permits air to flow through vents in the tip until it conforms to the shape of the cornea. Once the force of pressure being applied to the cornea is equal to the force of pressure in the anterior chamber, the pneumatic sensor charts and records both IOP and an ocular pulse waveform (Figure 2C). Each dog went through this measurement process twice, once to become acclimated to the probe and noise of the machine and a second time for data collection. The data were recorded and printed via the pneumotonometer’s integrated chart strip printout.

Lastly, dogs received one drop of OptixCare^®^ Plus Eye Lube (CLC MEDICA, Ontario, Canada) and were placed in a seated position for central corneal thickness (**CCT**) measurements taken via PachPen^®^ handheld pachymeter (Accutome, Inc., Malvern, PA, USA). Dogs were also in a seated position while the axial length (**AXL**) of the eye was measured via Scanmate A DGH 6000 A-scan (DGH Technology, Inc., Exton, PA, United States).

Archival corneal diameter (CD) was available for 34/101 eyes (34/53 dogs). Measurements occurred using Jameson calipers (Integra Miltex, York, PA, United States). Estimated cumulative IOP (cumIOP) data were available for 81/101 eyes (43/53 dogs): Daily averages of archival diurnal IOPs measured with the iCare TonoVet^®^ rebound tonometer were plotted as a function of time in days. Areas under the curve were calculated to obtain estimates of cumIOPs (mmHg x days). Since IOP measurements were not performed continuously, the cumIOP data are considered estimates.

### Repeatability

A separate experiment was performed to determine the repeatability of OPA measurements in dogs. The same experimental setup was used as described for the main experiment. The left or right eye was randomly selected for every dog to begin measurements for OPA. Each of the 18 dogs went through the measurement process twice. The median interval between the first and second measurements was 19 min (range: 6–22 min).

### Statistical analyses

All outcome measures of left and right eyes were averaged for each dog. Data were analyzed by descriptive statistics, analysis of variance (ANOVA), regression analysis, and mixed effects models using R. ANOVA models were used to assess the effect of mutation on OPA and the effect of mutation by age on OPA. A linear model was applied to determine the impact of age on OPA. An analysis of covariance (ANCOVA) was used to analyze the effect of mutation by age on OPA with the following control variables: IOP, PP, AXL, CCT, VPA, CD, and cumIOP. These analyses were performed for all dogs combined as well as by excluding young adult dogs <12 months of age. Possible relationships between variables were evaluated by bivariate correlation analysis for all dogs combined but also separately for normal and *ADAMTS10*-mutant dogs. Pearson correlation coefficient was calculated between age and AXL for the normal and *ADAMTS10*-mutant groups. A Bland-Altman analysis was used to interpret the repeatability data.

Intraocular pressure measured by iCare TonoVet^®^ rebound tonometer was used for all the analyses. Even though not the primary purpose of this study, IOPs measured by iCare TonoVet^®^ and Reichert Model 30™ Pneumotonometer were compared with Bland-Altman plots and by regression analysis without averaging left and right eyes (Prism 9; GraphPad Software, Boston, MA).

## Results

### Effect of *ADAMTS10* mutation on OPA

**FIGURE 3:**
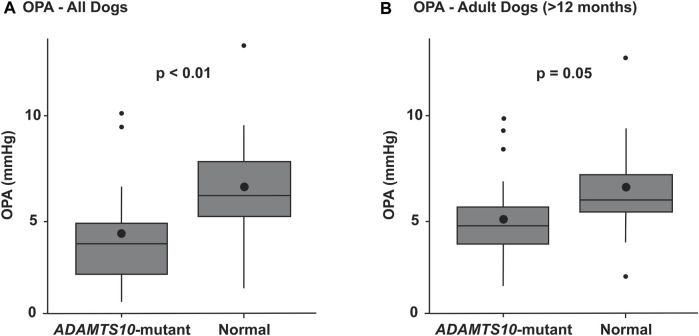
Box plots summarizing the OPAs of *ADAMTS10*-mutant and normal dogs. **(A)** The mutant group had a significantly smaller OPA than the normal control group (*p* < 0.01). **(B)** When excluding the younger dogs <12 months, the same trend remained, but the difference was no longer significant (*p* = 0.05). The boxes represent the middle 50% of the data, with the centerline being the median. The bottom and top whiskers show the first and third quartiles, respectively. Outliers are shown as small black dots. The larger black dots inside the boxes represent the means.

**FIGURE 4:**
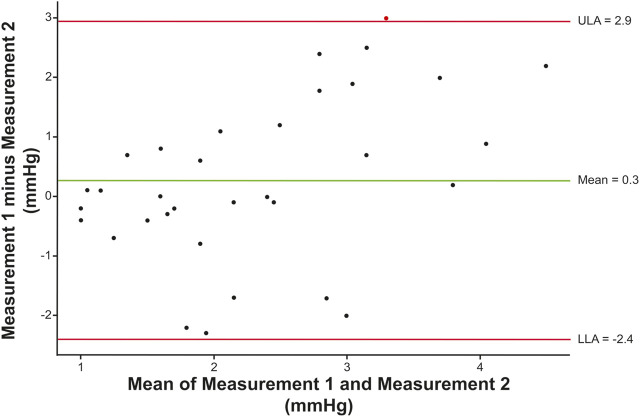
Repeatability of canine OPA measurements. The Bland-Altman plot compares first and second measurements in a group of 34 eyes of 18 dogs (median time interval: 19 min, range: 6–22 min). The estimated means of the differences between the two measurements are shown by the green horizontal line. The 95% CI is marked by the red lines representing the upper (ULA) and lower (LLA) limits of agreement (mean ± 1.96 SD). Overall, the OPA measurements did not follow any trends and were mainly located within the 95% CI.

Ocular pulse amplitude could be easily measured in lightly sedated dogs. Because there was a significant correlation between left and right eyes on OPA (r = 0.42, 95% CI [0.16, 0.63], *p* = 0.003), outcome measures were averaged for each dog. Testing by ANOVA revealed that OPA was significantly lower in *ADAMTS10*-mutant dogs (4.1 ± 2.0 mmHg; mean ± standard deviation) compared to normal control dogs (6.5 ± 2.8 mmHg; *p* < 0.01; Figure 3A). When controlling for the following variables, the mutation effect became even more significant (*p* < 0.001): age, mutation by age, IOP, cumIOP, PP, AXL, CCT, CD, and gender. With 39 *ADAMTS10*-mutant and 14 normal dogs, a desired 0.05 type I error, and a power of 0.80 the detectable effect size was considered reasonable with 0.89 standard deviations (SD). When excluding the younger dogs <12 months, the same trend remained, although the difference in OPA was no longer significant (mutant vs. normal: 4.9 ± 2.2 mmHg vs. 6.5 ± 3.0 mmHg; *p* = 0.05; Figure 3B). In a separate repeatability study, the Bland-Altman plot showed good agreement between first and second OPA measurements (Figure 4).

As expected, IOPs were significantly higher in mutant vs. normal dogs (23.0 ± 7.0 mmHg vs. 15.3 ± 3.6 mmHg; *p* < 0.0001), but PPs were not significantly different despite being smaller in mutant dogs (75.5 ± 20.9 mmHg vs. 88.6 ± 29.7 mmHg; *p* = 0.18). Axial length was significantly larger in mutant vs. normal dogs (20.9 ± 0.6 mm vs. 19.8 ± 0.8 mm; *p* = 0.01), but there was no significant difference in mean CCT (666.3 ± 52.0 μm vs. 634.1 ± 24.7 μm; *p* = 0.74) between mutant and normal dogs. There was no significant difference in CD, regardless of whether young dogs <12 months were included (mutant vs. normal 15.9 ± 0.9 mm vs. 15.3 ± 0.6 mm) or not (mutant vs. normal 16.1 ± 1.0 vs. 15.3 ± 0.6 mm). The difference in AXL remained when young dogs <12 months were excluded (21.2 ± 0.5 mm vs. 20.1 ± 0.6 mm; *p* < 0.001). We could not detect a significant difference in cumIOP between *ADAMTS10*-mutant and normal dogs (17,058 ± 14,309 mmHg day vs. 14,981 ± 8,280 mmHg day; *p* > 0.9).

### Other parameters affecting OPA

**FIGURE 5:**
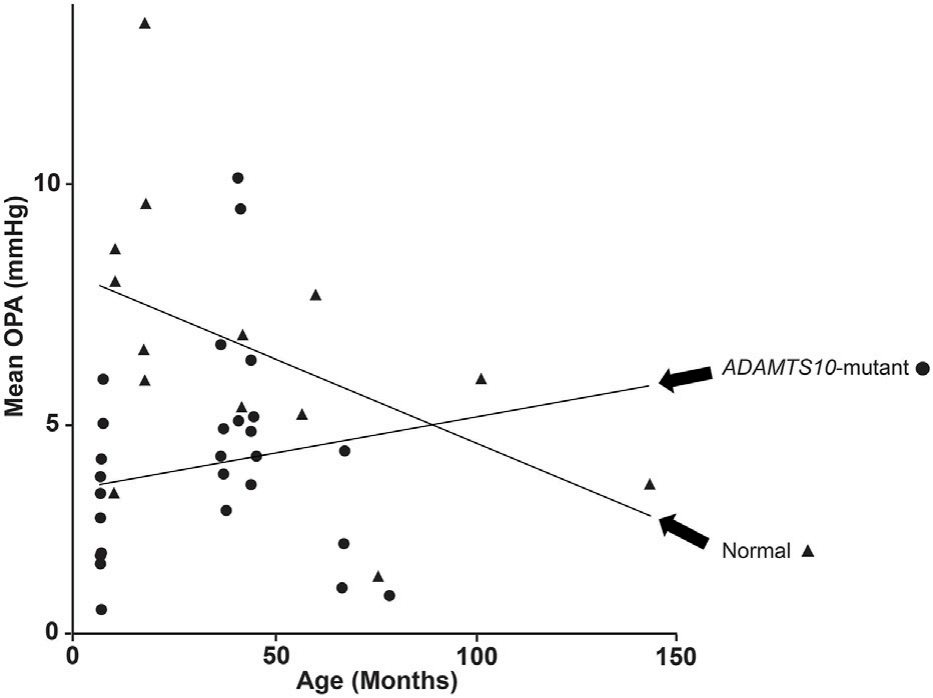
Change of OPA with age for *ADAMTS10*-mutant and normal dogs. Despite the trendlines, there was no detectable age effect on OPA.

**FIGURE 6:**
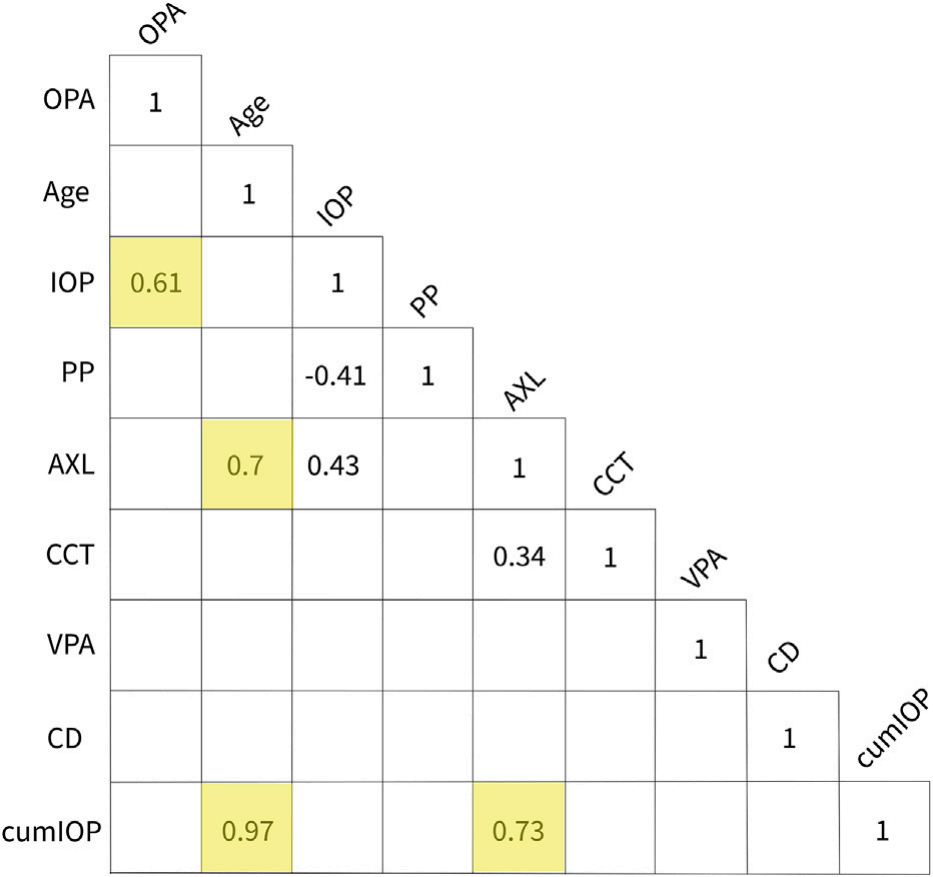
Summary of correlations. Unshaded numbers show that OPA was not correlated with any of the other parameters when analyzing all dogs together. Significant correlations were found between IOP and PP, IOP and AXL, and AXL and CCT. No significant correlations were found in the normal dog group. The yellow-shaded numbers show the significant correlations in the *ADAMTS10*-mutant dog group: These were between OPA and IOP, age and AXL, age and cumIOP, and between AXL and cumIOP. Abbreviations: AXL, axial length; CD, corneal diameter; cumIOP, cumulative IOP; IOP, intraocular pressure; OPA, ocular pulse amplitude; CCT, central corneal thickness; PP, perfusion pressure; VPA, vascular pulse amplitude.

There was no OPA age effect over all the dogs tested (Figure 5). The age range for normal dogs was 11 years (133 months), and the median was 2.5 years (30 months). The age range for *ADAMTS10*-mutant dogs was 6 years (73 months), and the median was 3 years (38 months) (Figure 1). When tested over all dogs, we did not find any significant correlations between OPA and these parameters: IOP (*p* = 0.17), PP (*p* = 0.34), AXL, (*p* = 0.62), CCT (*p* = 0.91), and VPA (*p* = 0.34) (Figure 6). We did find significant correlations between IOP and PP (r = −0.41; *p* = 0.01), IOP and AXL (r = 0.43; *p* = 0.001), and AXL and CCT (r = 0.34; *p* = 0.02). When analyzing *ADAMTS10*-mutant dogs alone, we found a significant positive correlation between OPA and IOP (r = 0.61; *p* = 0.004) (Figure 6). Furthermore, mutant dogs showed significant positive correlations between age and AXL (r = 0.7; *p* < 0.001), between age and cumIOP (r = 0.97; *p* < 0.001), and between AXL and cumIOP (r = 0.73; *p* < 0.001). There were no significant correlations between any of the parameters in the normal control dogs, including age and AXL (Figure 6).

### Comparison of tonometers

**FIGURE 7:**
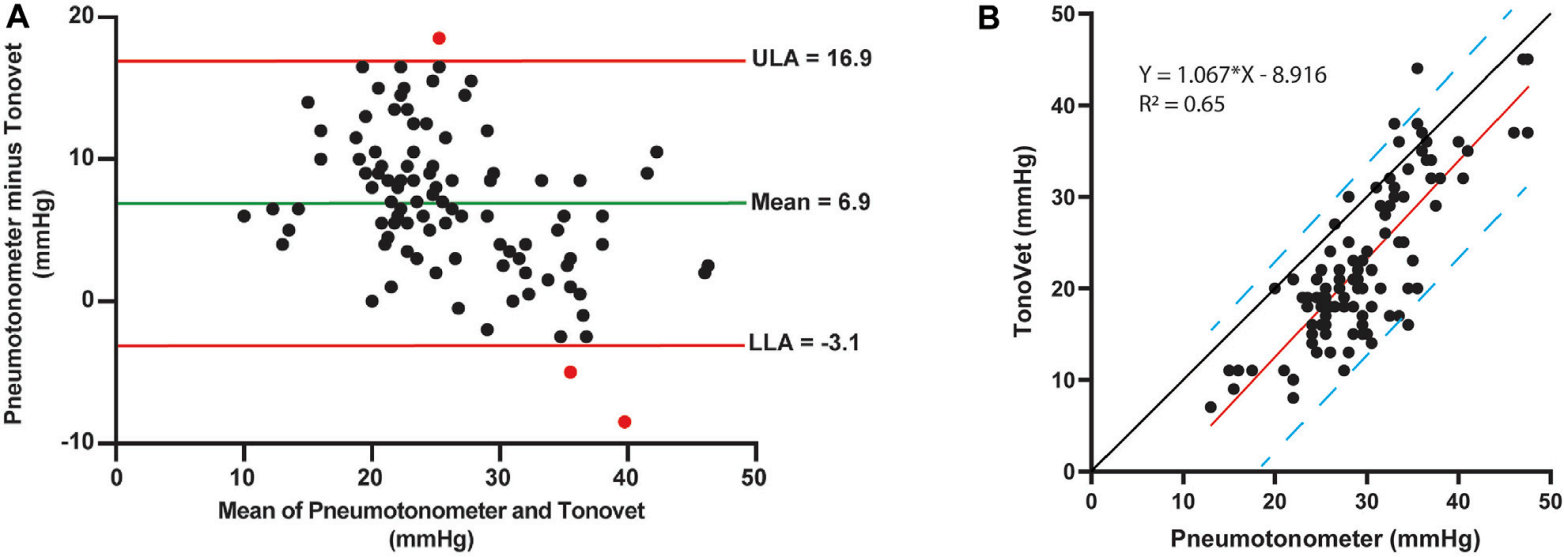
Comparison of tonometers. **(A)** Bland-Altman plot comparing Reichert Model 30™ pneumotonometer and iCare TonoVet^®^ rebound tonometer. The estimated means of the differences between the two tonometers are shown by the green horizontal line. The 95% CI is marked by the red lines representing the upper (ULA) and lower (LLA) limits of agreement (mean ± 1.96 SD). Overall, the IOP measurements did not follow any trends and were mainly located within the 95% CI. The estimated means of the differences (±SD) were 6.9 ± 5.1 mmHg. **(B)** IOP regression analyses between tonometers with linear equation and *R* ^2^ values. The red line shows strong linear correlation between Reichert Model 30™ pneumotonometer and iCare TonoVet^®^ rebound tonometer with the blue dashed lines marking the 95% prediction intervals. Compared to the black y = x line, the pneumotonometer overestimated most IOPs when compared to the TonoVet^®^ with data points mostly located to the left of the y = x line.

Because the iCare TonoVet^®^ is the standard tonometer used in our laboratory and widely used in veterinary clinical practice, we used IOP measurements taken with this instrument for our statistical analyses. However, as part of the study design, IOPs were also measured with the Reichert Model 30™ pneumotonometer immediately before OPA assessment; these readings were not used in our analyses. Nevertheless, Bland-Altman plot and linear regression analysis showed good agreement between the two tonometers with the pneumotonometer overestimating most IOPs compared to the TonoVet® (Figure 7).

## Discussion

Ocular stiffness may be essential to glaucoma pathophysiology, but the exact role of tissue biomechanics and susceptibility to IOP-related damage is not entirely understood (Park and Komaromy, 2021; Safa et al., 2022). The most commonly used clinical assessments of ocular biomechanics are limited to the cornea and consist of CCT and corneal hysteresis (Gordon et al., 2002; Medeiros et al., 2013; Ayyalasomayajula et al., 2016; Weinreb et al., 2016). Because of their importance in IOP susceptibility, there is a critical need for additional clinical tools that include the biomechanics of the PPS and lamina cribrosa (Downs et al., 2009; Burgoyne, 2011; Safa et al., 2022). Measurement of OPA may be such a tool. The extent of transient IOP fluctuations, such as from saccades, blinking, ocular pulse, or controlled microvolumetric intraocular injections, is related to overall ocular coat stiffness, including corneal and PPS stiffness (Liu and He, 2009; Morris et al., 2013; Beaton et al., 2015; Sturmer and Kniestedt, 2015; Clayson et al., 2017; Sherwood et al., 2019; Turner et al., 2019; Sayah et al., 2020; Ma et al., 2021; Markert et al., 2022). In the current study we demonstrated that compared to normals the OPA is significantly reduced in *ADAMTS10*-mutant Beagles, a well-established, clinically relevant large animal model of OAG with decreased scleral stiffness and normalized ocular rigidity (Palko et al., 2013; Boote et al., 2016; Palko et al., 2016). We suspect that the softer *ADAMTS10*-mutant sclera is in part responsible for an improved IOP tolerance with slower progression of ONH atrophy and vision loss in Beagles with *ADAMTS10*-OAG compared to other glaucoma-affected canine breeds with comparable IOPs (Kuchtey et al., 2011; Park and Komaromy, 2021).

Ocular pulse amplitude is the difference between diastolic and systolic IOP and is created by the pulsatile choroidal blood flow (Dastiridou et al., 2009; Beaton et al., 2015; Sturmer and Kniestedt, 2015). Our conclusions on *ADAMTS10*-mutant dogs are based on observations in human patients that OPA and ocular rigidity are correlated at controlled IOPs of 15–45 mmHg: increased transient IOP fluctuations, such as OPA, are associated with greater rigidity (Dastiridou et al., 2009). However, the relationship between OPA and ocular rigidity may differ for different eyes because many other factors impact OPA. The OPA measurements using a pneumotonograph were well tolerated and easily performed in lightly sedated dogs, supporting the potential routine clinical application. The reasonable repeatability of the canine OPA measurements demonstrated in a separate experiment further supports their potential clinical usefulness.

Our canine OPA values (normal: 6.5 ± 2.8 mmHg; *ADAMTS10*-mutant: 4.1 ± 2.0 mmHg) are comparable to the 0.9–7.2-mmHg range in normal and glaucomatous humans; although the mean OPAs in dogs are lower than previously reported in humans (2.2 and 3.0 mmHg, respectively) (Kaufmann et al., 2006; Kac et al., 2011; Sturmer and Kniestedt, 2015). The higher pressure readings by the pneumotonometer could explain this. The obtained data supported our hypothesis that the softer *ADAMTS10*-mutant sclera may be partly responsible for the significantly lower OPA because it provides less resistance to the pulsatile choroidal blood flow. Our finding of lower OPA in *ADAMTS10*-mutant dogs is even more significant, considering that their ocular rigidity and OPA should be higher with higher baseline IOPs (Friedenwald, 1937; Dastiridou et al., 2009; Downs, 2015).

While not widely used in clinical settings, OPA was measured previously in humans with different forms of glaucoma; however, IOP susceptibility was not explicitly studied. In most studies, OPA with chronic POAG was not different from normal individuals (Sturmer and Kniestedt, 2015). Some studies showed reduced or elevated OPA in POAG patients (Trew and Smith, 1991a; Trew and Smith, 1991b; Mittag et al., 1994; Schmidt et al., 1997; Kerr et al., 1998; Schmidt et al., 1998; Khan et al., 2002; Kniestedt et al., 2006; Punjabi et al., 2006; Romppainen et al., 2007; Stalmans et al., 2008; Vulsteke et al., 2008; Asejczyk-Widlicka et al., 2014; Katsimpris et al., 2014; Sturmer and Kniestedt, 2015; Milioti et al., 2017). These variations were based on diverse study populations with various degrees of glaucomatous optic neuropathy, different IOP ranges, and the use of IOP-lowering medications (Sturmer and Kniestedt, 2015). In general, OPA increased with progressing POAG, suggesting increased ocular stiffening (Romppainen et al., 2007; Weizer et al., 2007; Kac et al., 2011; Kynigopoulos et al., 2012; Moghimi et al., 2013; Gross et al., 2014; Sturmer and Kniestedt, 2015). Our OPA data supported this: differences between *ADAMTS10*-mutant and normal dogs became less significant when excluding young dogs before the development of glaucoma. Others have shown increased OPA with decreased glaucoma severity or better IOP control and decreased OPA with progressive ONH atrophy and visual field loss (Agarwal et al., 2003; Weizer et al., 2007; Vulsteke et al., 2008; Sturmer and Kniestedt, 2015).

Controversial results have also been reported when comparing NTG patients to normal: Some found that NTG patients had lower OPA or pulsatile ocular blood flow (**POBF**) than normal controls (Quaranta et al., 1994; Schmidt et al., 1997; Schwenn et al., 2002). Considering that NTG patients are more susceptible to IOP since they develop glaucomatous optic neuropathy and visual field loss without documented IOP elevation, these findings contradict our results with lower OPA in *ADAMTS10*-mutant dogs that are more resistant to IOP elevation. Others did not find OPA differences between NTG patients, normals, or chronic POAG-affected individuals (Khan et al., 2002; Kniestedt et al., 2006; Read et al., 2008; Stalmans et al., 2008; Vulsteke et al., 2008). Another study found significantly lower OPA in NTG than in POAG patients, OHT, and normal controls. A logistic regression model was established to identify OPA as an independent risk factor for NTG (Schwenn et al., 2002). OPA and IOP were significantly higher in NTG patients with central visual field loss compared to NTG patients without visual field loss, with a significant correlation between OPA increase and central visual field loss (Lee et al., 2012). OPA measurement could be helpful in the detection and screening of NTG patients, but additional studies are needed for validation.

In contrast to NTG patients, individuals with OHT are more resistant to IOP elevation without developing ONH damage (Weinreb et al., 2016). Studies of OPA in OHT-affected individuals are variable, with some showing higher ocular rigidity compared to POAG patients and others not showing an OPA difference between OHT, POAG, and control groups (Schwenn et al., 2002; Wang et al., 2013). In patients with OHT and chronic POAG, increased OPA was a significant risk factor for visual field loss (Gross et al., 2014).

While we did not include dogs treated with glaucoma medications, a weakness of several referenced human clinical studies is the unknown contribution of glaucoma treatments on the OPA. There are only limited data on the effect of topical glaucoma medications: For example, while the prostaglandin analog latanoprost and the carbonic anhydrase inhibitor dorzolamide HCl were associated with increased POBF, the beta-adrenergic blocker timolol maleate did not affect POBF (Claridge and Smith, 1994; Schmidt et al., 1998; Georgopoulos et al., 2002). Another study showed an OPA-lowering effect of the prostaglandin analogs tafluprost and latanoprost in human patients with NTG and POAG (Park et al., 2015).

The measurement of OPA is a reasonably old method and has been performed by several investigators over the past century on humans and various animal species (Thiel, 1928; Susuki, 1962; Bynke and Krakau, 1964; Lawrence and Schlegel, 1966; Lester, 1966; Bynke, 1968). The reliability of ocular pulse pressure waves measured with a pneumatic tonometer has been previously documented in dogs, confirmed with manometric systems, and compared to humans and rabbits (Tonjum, 1972). The pneumotonography used in this study has been well-established in dogs and is routinely used in our laboratory to measure aqueous humor outflow facility (Gelatt et al., 1996). The pneumotonography tonometer was previously compared to other applanation tonometers in dogs (Gelatt and MacKay, 1998). We have shown an excellent linear correlation between the Reichert Model 30™ Pneumotonometer and the iCare TonoVet^®^ rebound tonometer, with the pneumotonometer reading consistently higher than the rebound tonometer. Because the iCare TonoVet^®^ is the standard tonometer used in our laboratory and widely used in veterinary clinical practice, we used IOP measurements taken with this rebound tonometer for our statistical analyses.

As expected, IOPs were significantly higher in mutant vs. normal dogs because the G661R missense mutation in *ADAMTS10* is responsible for OAG (Kuchtey et al., 2011). The IOPs were not high enough to affect PPs significantly, although there was a significant negative correlation between IOP and PP. There were also no significant differences in CCT, but we found a significant correlation between IOP and CCT. In human glaucoma patients, OPA has been previously positively associated with CCT (Weizer et al., 2007). Axial length was significantly larger in our mutant vs. normal dogs, with a significant positive correlation between IOP and AXL, consistent with the chronic IOP increase associated with *ADAMTS10*-OAG and the development of buphthalmos. The significant linear age-AXL correlation in *ADAMTS10*-mutant dogs further supported this. Since there was no such correlation in normal dogs, and all enrolled canine eyes were considered adult-size (Tuntivanich et al., 2007), the increase in AXL with age was likely disease-related.

Despite the extensive age range (normal = 11 years; mutant = 6 years), we could not detect a significant OPA age effect in mutant and normal dogs. The trend lines in Figure 5 are likely affected by the outliers. We have previously reported that age was positively associated with complex modulus and negatively associated with loss tangent, suggesting increased stiffness and decreased mechanical damping with age in *ADAMTS10*-mutant and normal dogs (Palko et al., 2016). This disagreement should be investigated in a future study by comparing OPA and scleral biomechanics in the same canine globes–this was beyond the scope of the current, non-terminal study. We did observe that OPA differences became less significant when excluding young adult dogs <12 months of age.

We found a significant positive correlation between OPA and IOP in *ADAMTS10*-mutant dogs. This finding is consistent with several human studies that found positive correlations between OPA and IOP in healthy individuals and patients with NTG and OHT (Phillips et al., 1992; Kaufmann et al., 2006; Kim et al., 2013; Sturmer and Kniestedt, 2015).

The remodeling of the sclera is likely more affected by cumIOP rather than a single measurement taken on the procedure day. Therefore, we attempted to include cumIOP as a variable. Because the number of archival IOP data varied between dogs and was not collected continuously, the cumIOP numbers in this study should be considered estimates. The numbers were highly variable and surprisingly not significantly different between mutant and normal dogs, possibly because of the more advanced age of some normal dogs. While we did not find any cumIOP-OPA correlation, age and AXL were positively correlated with cumIOP in *ADAMTS10*-mutant dogs.

The lack of a significant correlation between OPA and PP is consistent with results from human studies (Sturmer and Kniestedt, 2015). We also did not find a significant correlation between OPA and AXL and between OPA and CD. Previous studies reported significant negative correlations between OPA and AXL, especially in human eyes with high myopia (Kaufmann et al., 2006; Sturmer and Kniestedt, 2015; Yang and Koh, 2015).

We found no significant correlations between OPA and VPA in our dogs. One study in human NTG patients showed a positive correlation between OPA and blood pressure amplitude (Kim et al., 2013). The OPA and blood pressure variances ratio was a potential diagnostic indicator for early human POAG, but the study included only ten normal and 11 glaucoma patients (Robert et al., 2012).

The significant correlation between the two eyes’ OPA justified averaging parameters from the left and right eyes. A previous simulation study showed that using the average of both eyes of the same individual as an analysis unit preserved type I error better than other statistical techniques while maintaining the power (Huang et al., 2018).

In addition to the lack of direct comparison of OPA and scleral biomechanics in the same eyes, our study has several other limitations, including the relatively small sample size compared to most referenced human studies. Because we do not have any clear indication of reduced ocular blood flow in *ADAMTS10*-mutant dogs, the additional measurement of pulsatile blood flow would have strengthened the main conclusions of our study regarding the OPA-scleral biomechanics relationship. Nevertheless, to our knowledge, this is the first study that evaluates OPA as a potential clinical measure for ocular rigidity in a well-established large animal glaucoma model with well-defined scleral biomechanics. Others proposed a relationship between corneal hysteresis and OPA and their heritability in human subjects (Kaufmann et al., 2006; Carbonaro et al., 2008). Future studies will be strengthened by directly comparing OPA with *ex vivo* corneal and scleral biomechanical measurements in the same eyes. We are aware of the potential limitations of OPA measurement since it is not only influenced by ocular rigidity but also by other factors, such as the amplitude of the pulsatile blood flow. Nevertheless, the expanded clinical testing of ocular rigidity by including OPA has excellent potential in assessing glaucoma risk relative to IOP.

## Data Availability

The raw data supporting the conclusions of this article will be made available by the authors, without undue reservation.

## Abbreviations

*ADAMTS10*, A disintegrin and metalloproteinase with thrombospondin motifs 10; ANCOVA, analysis of covariance; ANOVA, analysis of variance; AXL, axial length; CCT, central corneal thickness; CD, corneal diameter; cumIOP, cumulative IOP; IOP, intraocular pressure; NTG, normal tension glaucoma; OAG, open-angle glaucoma; OHT, ocular hypertension; OPA, ocular pulse amplitude; ORA, Ocular Response Analyzer^®^; POAG, primary open-angle glaucoma; POBF, pulsatile ocular blood flow; PP, perfusion pressure; PPS, peripapillary sclera; VPA, vascular pulse amplitude.
